# Prevalence and Main Clinical Characteristics of Fully Vaccinated Patients Admitted to Hospital for Delta Variant COVID-19

**DOI:** 10.3389/fmed.2022.809154

**Published:** 2022-03-02

**Authors:** Matteo Vassallo, Nicolas Clement, Laurene Lotte, Sabrina Manni, Audrey Sindt, Pierre M. Bertrand, Jacques Durant

**Affiliations:** ^1^Department of Internal Medicine/Infectious Diseases, Cannes General Hospital, Cannes, France; ^2^Intensive Care Unit, Cannes General Hospital, Cannes, France; ^3^Multipurpose Laboratory, Bacteriology and Virology Unit, Cannes General Hospital, Cannes, France; ^4^Department of Infectious Diseases, Nice University Hospital, Nice, France

**Keywords:** COVID-19, delta variant, vaccination, patients, hospitalization

## Abstract

**Objectives:**

The Delta variant of the novel beta coronavirus responsible for the current coronavirus pandemic (COVID-19) spread across Europe during the summer of 2021. Little is known of vaccine efficacy on this variant. Our aim was to study the prevalence and clinical characteristics of fully vaccinated subjects admitted to hospital for Delta variant COVID-19.

**Methods:**

We identified patients admitted to Cannes hospital for Delta-variant-related Covid-19 infection from July to September 2021. Their main demographic parameters, inflammatory markers, and clinical characteristics were recorded. Differences between fully vaccinated subjects and unvaccinated or incompletely vaccinated individuals were analyzed.

**Results:**

We included 126 patients (57% male, mean age 64 years, mean delay since symptoms onset 7.8 days). Among admitted patients, 94 (75%) were not vaccinated, 11 (8%) incompletely so and 21 (17%) were fully vaccinated. Fully vaccinated patients were older (77 vs. 61 vs. 62 years, *p* = 0.003), with fewer days since symptoms onset (5.9 vs. 8.0 vs. 9.3 days, *p* = 0.035) than unvaccinated or incompletely vaccinated patients, respectively. Severe pneumonia was less frequent among completely vaccinated subjects (67 vs. 84 vs. 100%, *p* = 0.038), while rates of transfer to the ICU, mechanical ventilation or death did not differ. Thirteen fully vaccinated patients underwent a thoracic CT scan, revealing involvement of lung parenchyma in four of them.

**Discussion:**

Prevalence of hospitalization for Delta-variant COVID-19 in fully vaccinated subjects was low and, despite their age and comorbid conditions, these patients had a high rate of favorable outcome.

## Introduction

SARS-Cov-2 is a betacoronavirus first reported in Wuhan, China, in December 2019 (1–3). It rapidly spread worldwide, resulting in the current COVID-19 pandemic.

Several genetic variants of concern (VOC) emerged as a consequence of acquired mutations during viral replication. In Europe, the alpha variant (20I/501Y.V1) became rapidly prevalent across Europe in early 2021 and has been responsible for a major increase in hospital admissions in several countries, including France (4–7). It has been now replaced by the novel and more contagious B.1.617.2 strain, also named Delta variant, first reported in India and now responsible for almost all cases of COVID-19 in Europe (8).

Highly efficacious vaccines were introduced in December 2020, with a significant impact on hospitalizations and death (9–11). Using different techniques, vaccines generate a potent immune response against the virus' spike protein, which initiates host cell infection by binding with Angiotensin-converting enzyme 2 (ACE2) (12–14).

As VOCs could harbor mutations within the spike protein, a potential reduction in vaccine protection may be feared. While the efficacy of vaccines against the original strain and the alpha variant has been largely proved (15–17), data on the Delta variant are scarcer. *In vitro* data demonstrate efficacy of most vaccines on the Delta variant, but several cases of Covid infection despite full vaccination have been described (18, 19). Indeed, current vaccines are administered intramuscularly or intradermally; they induce mainly systemic IgG antibody response and cellular T-cell immunity, generating a potent protection against infection of the lower respiratory tract. However, the production of IgA antibodies, which confers mucosal immunity and consequently protection of the upper respiratory tract, is generally low with these vaccines. Current vaccines thus mainly induce disease-preventing or disease-attenuating, but not necessarily sterilizing, immunity (20).

Most cases of COVID-19 despite full vaccination have been described among outpatients (18). The aim of this study was to evaluate the prevalence of hospital admissions for Delta variant COVID-19 despite complete vaccination, and to describe patients' main characteristics and clinical outcome.

## Methods

### Study Design and Participants

We conducted an observational, retrospective cohort study of patients admitted to the Department of Internal Medicine or Intensive Care Unit in Cannes General Hospital, with confirmed delta variant COVID-19 infection, between July 15 and September 10, 2021, when our region faced a major new outbreak of COVID-19 infection.

Data from each patient were extracted from the hospital electronic database. Patients with positive delta variant strain SARS-CoV-2 RT-PCR, detected in nasal swabs and screening samples were included.

Patient demographics, underlying comorbidities, duration of symptoms, clinical signs prior to admission, upon admission and during hospitalization, laboratory findings during hospital stay, management and clinical outcome were collected from their medical records.

In line with WHO criteria for COVID-19 clinical severity (21), patients were divided into the following groups:

- Mild to moderate disease (mild symptoms up to mild pneumonia).
- Severe disease (dyspnea, hypoxia or over 50% lung involvement as shown on the CT scan).

COVID-19 vaccination status, type, and date of vaccination were also collected. According to their vaccine status, patients were divided into three groups:

- Those either not vaccinated.
- Those with incomplete vaccination.
- Those fully vaccinated.

Subjects were considered fully vaccinated in case of hospital admission at least 7 days following the second injection, with the exception of the Johnson and Johnson vaccine, for which complete vaccination was defined as an interval of 28 days since the injection. According to French guidelines, in case of previous COVID-19 illness, subjects with just one dose of vaccine were also considered fully vaccinated (22).

Files were reviewed directly by the physicians who had cared for the patients during their hospital stay.

After a comparison between vaccinated, incompletely vaccinated and unvaccinated subjects, we focused our analysis on vaccinated patients, detailing their main clinical characteristics, together with laboratory and imaging results.

### Laboratory Markers

The following biological markers were included in the statistical analysis: C-reactive protein, white cell, neutrophil, and platelet counts, hemoglobin, D-dimer and IL-6 (electrochemiluminescence immunoassay, Cobas^®^ 6000 Roche, pg/ml). RT-PCR assays were either performed in the multipurpose laboratory of Cannes General Hospital or in the Bioesterel Biogroup laboratory. According to the study period and laboratory guidelines, four RT-PCR assays were used: the NeuMoDX^®^ (Qiagen) instrument (targeting N and NSP2 genes), the GeneXpert^®^ (Cepheid, E and N2 genes), the Gene Finder COVID-19 Plus RealAmp kit (GeneFinder, N, E, and RdRP genes) or the SARS-Cov-2 ELITE MGB kit (Elitech, ORF8, and RdRP genes). Virus isolates were screened to identify N501Y, E484K, and K417N mutations, using GSD NovaType II SARS CoV-2 and NovaTec Immundiagnostica GmbH assay.

### Statistical Analysis

Categorical variables were described as frequency rates and percentages, while continuous variables were detailed with mean, median, and inter-quantile range (IQR).

Fully vaccinated patients were compared with those either not vaccinated or incompletely vaccinated.

χ^2^-tests and ANOVA-tests were performed to compare variables, and independent risk factors were identified by multivariate analysis.

Variables with a *p* ≤ 0.20 in univariate analysis were initially selected for the multivariate model and only those with a *p* ≤ 0.05 were retained in the final model. All analyses were performed using Statview software.

## Results

### Study Population

From July 15 to September 15, 2021, we identified 126 subjects admitted to the hospital for delta variant COVID-19 (57% male, mean age 64 years, mean delay since symptoms onset 7.8 days). Thirty-three patients (26%) required transfer to the ICU, while the mortality rate was 15%. Among the 126 patients, 94 (75%) were unvaccinated, 11 (8%) were incompletely vaccinated and 21 (17%) were fully vaccinated against COVID-19.

Rates of hypertension and severe neurocognitive disorders were higher among fully vaccinated subjects (Table 1).

**Table 1:** Characteristics of patients admitted for COVID-19.

	**Not vaccinated**	**Incompletely vaccinated**	**Fully vaccinated**	***p*-value**
	***N* (%) or mean [SD]**	***N* (%) or mean [SD]**	***N* (%) or mean [SD]**	
**Number of patients**	94	11	21	
**Male gender**	53 (56%)	8 (73%)	11 (52%)	0.521
**Age (years)**	61.3 [19.7]	61.8 [16.0]	77.1 [14.3]	**0.003**
**Hypertension**	31 (33%)	3 (27%)	14 (67%)	**0.012**
**Diabetes**	19 (20%)	1 (9%)	8 (38%)	0.112
**Overweight**	23 (24%)	1 (9%)	4 (19%)	0.474
**Obesity**	25 (27%)	3 (27%)	4 (19%)	0.764
**Severe cognitive disorders**	3 (3%)	2 (18%)	4 (19%)	**0.013**
**Cancer**	3 (3%)	1 (9%)	2 (11%)	0.365
**Days of symptoms onset**	8.0 [3.9]	9.3 [4.9]	5.9 [3.2]	**0.035**
**Severe pneumonia**	79 (84%)	11 (100%)	14 (67%)	**0.038**
**Transfer in ICU**	25 (27%)	4 (36%)	4 (19%)	0.563
**Mechanical ventilation**	9 (10%)	1 (9%)	1 (5%)	0.778
**Death**	14 (15%)	2 (18%)	3 (14%)	0.953

*ICU, Intensive Care Unit. Values in bold were those with significant p values*.

### Characteristics of Fully Vaccinated Patients and Differences With Unvaccinated or Incompletely Vaccinated Individuals

Among the 21 fully vaccinated patients, 13 had received the Pfizer-Biontech, 3 the Moderna, 3 the AstraZeneca, and 2 the Johnson and Johnson formulations.

Fully vaccinated patients were older (77 vs. 61 vs. 62 years, *p* = 0.003), with fewer days since symptoms onset (5.9 vs. 8.0 vs. 9.3 days, *p* = 0.035) than unvaccinated or incompletely vaccinated patients, respectively. Severe pneumonia was less frequent among completely vaccinated subjects (67 vs. 84 vs. 100%, *p* = 0.038), while rates of transfer to the ICU, mechanical ventilation or death did not differ (Table 1).

All but one fully vaccinated subject had comorbid conditions. Mean interval between complete vaccination and hospital admission was 3.9 months (Table 2). Main comorbidities included hypertension, diabetes and overweight or obesity. Average duration of O_2_ therapy was <5 days. In 13 cases a thoracic CT scan was obtained during the hospital stay, and mean involvement of lung parenchyma was estimated at 30% (Table 2).

**Table 2:** Characteristics of fully vaccinated patients admitted to the hospital for COVID-19.

	***N* (%) or mean [SD]**
**Number of patients**	21
**Male gender**	11 (52%)
**Age (years)**	77 [14.3]
**Comorbid conditions**	
Hypertension	14 (67%)
Diabetes	8 (38%)
Overweight or obesity	8 (38%)
Neurocognitive disorders	4 (19%)
Cancer	2 (11%)
**Vaccine manufacturers**	
Pfizer	13 (62%)
Moderna	3 (14%)
AstraZeneca	3 (14%)
Johnson	2 (10%)
**Months between vaccination and hospital admission**	3.9 [1.9]
**Clinical characteristics**	
Severe pneumonia	14 (67%)
Mild to moderate disease	7 (33%)
Max O_2_ therapy > 3 l/min	12 (57%)
Days on O_2_ therapy	4.9 [5.6]
Transfer in Intensive Care Unit	4 (19%)
Mechanical ventilation required	1 (5%)
Death	3 (14%)
**Radiological characteristics**	
Mean percentage of lungs involvement at CT scan (*n* = 13)	30% [20]
**Laboratory markers at admission**	
Mean C-reactive protein (mg/l)	107 [23.4]
Absolute lymphocyte count (cc/mmcc)*	[527]

*One subject excluded for acute leukemia.

Laboratory markers showed increased C-reactive protein values and a low absolute lymphocyte count (Table 2).

Moreover, SARS-Cov2 viral loads, measured according to the RT-PCR cycle threshold values in nasal swabs with either NeuMoDX^®^ or GeneXpert^®^, did not differ between fully vaccinated subjects and those incompletely or not vaccinated (data not shown).

Three patients unfortunately died during their hospital stay. Two of them had been vaccinated with the AstraZeneca and one with the Johnson and Johnson formulations. One of them was a 77 year-old woman with lymphoma as the main comorbid condition. The likely cause of death was not related to COVID, but to acute leukemia.

The second patient was a 75 years-old man, hypertensive, diabetic, obese, with chronic ischemic cardiopathy as main comorbid conditions. He died from COVID-19-related acute respiratory distress syndrome (ARDS).

The third patient was a 90 years-old man known for chronic obstructive pulmonary disease. He developed an acute renal and heart failure during the hospital stay, and died probably from pulmonary edema.

## Discussion

Among our study population, patients who were fully vaccinated against COVID-19 accounted for a minority of hospital admissions during the delta variant outbreak. Indeed, about 85% of patients admitted for COVID-19 infection were either not vaccinated or incompletely vaccinated, and their mean age was lower than during the previous original strain and alpha variant waves (23, 24), which could explain the relatively low number of deaths and ICU transfers. Indeed, we previously found high rates of death or transfer to the ICU in case of alpha-variant COVID-19, but patients were significantly older than during the delta variant outbreak (24). Nevertheless, hospital admission of younger patients at present is concerning, and confirms the need for extending vaccination programs to all adult age groups.

Fully vaccinated patients were significantly older, with a high prevalence of comorbid conditions. However, severe cases were less frequent, mean duration of O_2_ therapy was <5 days, mean extent of radiological lung involvement was only 30%, and clinical outcome did not differ from younger non- or incompletely vaccinated subjects.

To our knowledge, this is one of the largest reported cohort of fully vaccinated patients admitted to the hospital for Delta variant COVID-19. Despite being an elderly population with a high rate of comorbid conditions, their clinical outcome was in majority favorable, with a generally rapid recovery, and a low mortality rate.

These results confirm the protection conferred by vaccines against severe forms of delta variant COVID-19 infection. Indeed, although several cases of illness despite full vaccination are currently described (25, 26), our study confirms that the risk of hospitalization is low, and the rate of favorable outcome is high. However, such results also emphasize the urgent need to understand the effect of waning neutralization titers and cellular immunity on protection against infection, especially that due to SARS-Cov-2 variants. Although the lack of neutralizing antibody levels before admission is a major limit, it is intriguing to note that the mean interval between complete vaccination and hospital admission was <4 months. Recent data showed that vaccine effectiveness against symptomatic disease decreased by 32% over a period of 6 months for subjects older than 50 years (27, 28). For such reasons, in order to improve the vaccine effectiveness, French guidelines recently reduced the suggested interval between the primary vaccination and the booster dose to 3 months (29).

Interestingly, viral load was similar in vaccinated and unvaccinated subjects. The degree of protection against virus transmission by vaccinated individuals compared to unvaccinated subjects remains a subject of debate (26, 30). Due to the limited number of patients with available cycle threshold values, and to the difference in duration of symptoms between the two groups, no conclusion can be drawn, and further studies are necessary. However, new vaccine platforms are currently being developed. Indeed, mucosal vaccines would probably improve protection against viral carriage by inducing a potent adaptative immunity at the mucosal site, rather than protecting against disease symptoms, such as that provided by currently available vaccines (31).

Limitations of this study include its retrospective character, the lack of neutralizing antibody levels before admission, and the relatively low number of patients. However, files were carefully reviewed by the same physicians who took care of the patients during their hospital stay.

In conclusion, few fully vaccinated patients were admitted for COVID-19 during the delta variant outbreak and, although these were mostly elderly with frequent comorbidities, their rates of favorable clinical outcome were high.

## Data Availability Statement

The original contributions presented in the study are included in the article/supplementary material, further inquiries can be directed to the corresponding author/s.

## Ethics Statement

Ethical Review and approval was not required for the study on human participants in accordance with the local legislation and institutional requirements. The patients/participants provided their written informed consent to participate in this study.

## Author Contributions

MV, NC, and LL: conceived, designed the study, and collected data. MV: analyzed the data and wrote the manuscript. SM, AS, PB, and JD: edited the manuscript. All authors contributed to the article and approved the submitted version.

## Conflict of Interest

The authors declare that the research was conducted in the absence of any commercial or financial relationships that could be construed as a potential conflict of interest.

## Publisher's Note

All claims expressed in this article are solely those of the authors and do not necessarily represent those of their affiliated organizations, or those of the publisher, the editors and the reviewers. Any product that may be evaluated in this article, or claim that may be made by its manufacturer, is not guaranteed or endorsed by the publisher.

## References

[1] Huang C Wang Y Li X Ren L Zhao J Hu. Clinical features of patients infected with 2019 novel coronavirus in Wuhan, China. Lancet. (2020) 395:497–506. 10.1016/S0140-6736(20)30183-5 31986264 PMC7159299

[2] Wang D Hu B Hu C Zhu F Liu X Zhang J. Clinical characteristics of 138 hospitalized patients with 2019 novel coronavirus-infected pneumonia in Wuhan, China. JAMA. (2020) 323:1061–9. 10.1001/jama.2020.1585 32031570 PMC7042881

[3] Chen Q Zheng Z Zhang C Zhang X Wu H Wang J. Clinical characteristics of 145 patients with corona virus disease 2019 (COVID-19) in Taizhou, Zhejiang, China. Infection. (2020) 28:1–9. 10.1007/s15010-020-01432-5 32342479 PMC7186187

[4] Gaymard A Bosetti P Feri A Destras G Enouf V Andronico A. Early assessment of diffusion and possible expansion of SARS-CoV-2 Lineage 20I/501Y.V1 (B.1.1.7, variant of concern 202012/01) in France, January to March 2021. Euro Surveill. (2021) 26:2100133. 10.2807/1560-7917.ES.2021.26.9.2100133 33663644 PMC7934223

[5] Leung K Shum MH Leung GM Lam TT Wu JT. Early transmissibility assessment of the N501Y mutant strains of SARS-CoV-2 in the United Kingdom, October to November 2020. Eurosurveillance. (2021) 26:2002106. 10.2807/1560-7917.ES.2020.26.1.2002106 33413740 PMC7791602

[6] Threat Assessment Brief: Rapid increase of a SARS-CoV-2 variant with multiple spike protein mutations observed in the United Kingdom. Eur Cent Dis Prev Control. (2020). Available online at: https://www.ecdc.europa.eu/sites/default/files/documents/Implicationsemergence-spread-SARS-CoV-2%20B.1.1.529-variant-concern-Omicron-for-the-EU-EEA-Nov2021.pdf

[7] Frampton D Rampling T Cross A Bailey H Heaney J Byott. Genomic characteristics and clinical effect of the emergent SARS-CoV-2 B.1.1.7 lineage in London, UK: a whole-genome sequencing and hospital-based cohort study. Lancet Infect Dis. (2021) 21:1246–56. 10.1016/S1473-3099(21)00170-5 33857406 PMC8041359

[8] SARS-COV-2 SARS-COV-2 Delta Variant Now Dominant in Much of the European Region Efforts Must Be Reinforced to Prevent Transmission Warn WHO/Europe, ECDC,. (2021). Available online at: https://www.ecdc.europa.eu/en/news-events/sars-cov-2-delta-variant-now-dominant-european-region (accessed October 15, 2021).

[9] US Food Drug Administration. Vaccines and Related Biological Products Advisory Committee Meeting: FDA Briefing Document. (2021). Available online at: https://www.fda.gov/advisory-committees/advisory-committee-calendar/vaccines-and-related-biological-products-advisory-committee-december-10-2020-meeting-announcement (accessed October 15, 2021).

[10] Polack FP Thomas SJ Kitchin N Absalon J Gurtman A Lockhart. Safety and efficacy of the BNT162b2 mRNA COVID-19 vaccine. N Engl J Med. (2020) 383:2603–15. 10.1056/NEJMoa2034577 33301246 PMC7745181

[11] Voysey M Clemens SAC Madhi SA Weckx LY Folegatti PM Aley K. Safety and efficacy of the ChAdOx1 nCoV-19 vaccine (AZD1222) against SARS-CoV-2: an interim analysis of four randomised controlled trials in Brazil, South Africa, the UK. Lancet. (2021) 397:99–111. 10.1016/S0140-6736(20)32661-1 33306989 PMC7723445

[12] Tortorici MA Beltramello M Lempp FA Pinto D Dang HV Laura Rosen E. Ultrapotent human antibodies protect against SARS-CoV-2 challenge *via* multiple mechanisms. Science. (2020) 370:950–7. 10.1126/science.abe3354 32972994 PMC7857395

[13] Letko M Marzi A Munster V. Functional assessment of cell entry and receptor usage for SARS-CoV-2 and other lineage B betacoronaviruses. Nat Microbiol. (2020) 5:562–9. 10.1038/s41564-020-0688-y 32094589 PMC7095430

[14] Wrapp D Wang N Corbett KS Goldsmith JA Hsieh CL Abiona O. Cryo-EM structure of the 2019-nCoV spike in the prefusion conformation. Science. (2020) 367:1260–3. 10.1126/science.abb2507 32075877 PMC7164637

[15] Egan C Thorpe M Knight S Shaw C McLean K Baillie K. Hospital Admission for COVID-19 Impact of Vaccination: Analysis of Linked Data From the National Immunisation Management Service (NIMS) the Coronavirus Clinical Information Network (CO-CIN). (2021). Available online at: https://assets.publishing.service.gov.uk/government/uploads/system/uploads/attachment_data/file/1018555/S1363_Hospital_Admission_for_COVID-19_and_impact_of_vaccination.pdf (accessed October 15, 2021).

[16] Bernal JL Andrews N Gower C Gallagher E Simmons R Thelwall S. Effectiveness of Covid-19 vaccines against the B.1.617.2 (Delta) variant. N Engl J Med. (2021) 385:585–94. 10.1056/NEJMoa2108891 34289274 PMC8314739

[17] Khoury DS Cromer D Reynaldi A Schlub TE Wheatley AK Jennifer A. Neutralizing antibody levels are highly predictive of immune protection from symptomatic SARS-CoV-2 infection. Nat Med. (2021) 27:1205–11. 10.1038/s41591-021-01377-8 34002089

[18] Fowlkes A Gaglani M Groover K Thiese MS Tyner H Ellingson K. Effectiveness of COVID-19 vaccines in preventing SARS-CoV-2 infection among frontline workers before and during B.1.617.2 (Delta) variant predominance - eight locations US, December 2020-August 2021. MMWR Morb Mortal Wkly Rep. (2021) 70:1167–9. 10.15585/mmwr.mm7034e4 34437521 PMC8389394

[19] Chen X Azman AS Lu W Sun R Zheng N Ge S. Prediction of vaccine efficacy of the Delta variant. medRxiv [Preprint]. (2021). 10.1101/2021.08.26.21262699 35086547 PMC8794738

[20] Krammer F. SARS-CoV-2 vaccines in development. Nature. (2020) 586:516–27. 10.1038/s41586-020-2798-3 32967006

[21] WHO Criteria for COVID-19 Clinical Severity. Available online at: https://www.who.int/publications/i/item/WHO-2019-nCoV-clinical-2021-1 (accessed October 15, 2021).

[22] Pass Sanitaire. Available online at: https://www.gouvernement.fr/info-coronavirus/pass-sanitaire (accessed October 15, 2021).

[23] Vassallo M Manni S Pini P Blanchouin E Ticchioni M Seitz-Polski B. Patients with Covid-19 exhibit different immunological profiles according to their clinical presentation. Int J Infect Dis. (2020) 101:174–9. 10.1016/j.ijid.2020.09.1438 33002623 PMC7521203

[24] Vassallo M Manni S Klotz C Fabre R Pini P Blanchouin E. Patients admitted for variant alpha COVID-19 have poorer outcomes than those infected with the old strain. J Clin Med. (2021) 10:3550. 10.3390/jcm10163550 34441844 PMC8396910

[25] Shastri J Parikh S Aggarwal V Agrawal S Chatterjee N Shah R. Severe SARS-CoV-2 breakthrough reinfection with delta variant after recovery from breakthrough infection by alpha variant in a fully vaccinated health worker. Front Med (Lausanne). (2021) 8:737007. 10.3389/fmed.2021.737007 34490316 PMC8418387

[26] Outbreak Outbreak of SARS-CoV-2 Infections Including COVID-19 Vaccine Breakthrough Infections Associated with Large Public Gatherings - Barnstable County Massachusetts July 2021. Available online at: https://www.cdc.gov/mmwr/volumes/70/wr/mm7031e2.htm?s_cid=mm7031e2_w (accessed October 15, 2021). 10.15585/mmwr.mm7031e2 PMC8367314 34351882

[27] Higdon MM Wahl R Jones CB Rosen JG Truelove SA Baidya A. A systematic review of COVID-19 vaccine efficacy and effectiveness against SARS-CoV-2 infection and disease. medRxiv [Preprint]. 10.1101/2021.09.17.21263549

[28] Goldberg Y Mandel M Bar-On Bar-On YM Bodenheimer O Freedman L Haas EJ. Waning immunity after the BNT162b2 vaccine in Israel. N Engl J Med. (2021) 385:e85. 10.1056/NEJMoa2114228 34706170 PMC8609604

[29] Avis n°. 0088/AC/SESPEV du 23 Décembre 2021 du Collège de la Haute Autorité de Santé Relatif à la Diminution du Délai Entre Primovaccination et Administration D'une Dose de Rappel et à L'administration D'une Dose de Rappel Chez Les Adolescents Fragiles Âgés de 12 à 17 Ans. (2021). Available online at: https://www.has-sante.fr/upload/docs/application/pdf/2021-12/avis_n_2021.0088.ac.sespev_du_23_decembre_2021_du_college_de_la_has_relatif_a_la_diminution_du_delai_e_2021-12-23_20-19-20_7.pdf (accessed December 30, 2021).

[30] Science Brief: COVID-19 Vaccines and Vaccination. Available online at: https://www.cdc.gov/coronavirus/2019-ncov/science/science-briefs/fully-vaccinated-people.html (accessed October 15, 2021).

[31] Lavelle EC Ward RW. Mucosal vaccines fortifying the frontiers. Nat Rev Immunol. (2021) 26:1–15. 10.1038/s41577-021-00583-2 34312520 PMC8312369

